# Hypermethylation of Cox5a Promoter Is Associated with Mitochondrial Dysfunction in Skeletal Muscle of High Fat Diet-Induced Insulin Resistant Rats

**DOI:** 10.1371/journal.pone.0113784

**Published:** 2014-12-01

**Authors:** Ying-ying Gong, Yuan-yuan Liu, Jin Li, Lei Su, Shuang Yu, Xiao-nan Zhu, Xiao-pei Cao, Hai-peng Xiao

**Affiliations:** 1 Department of Endocrinology, The First Affiliated Hospital of Sun Yat-sen University, Guangzhou, China; 2 Department of Geriatrics, The First Affiliated Hospital of Sun Yat-Sen University, Guangzhou, China; 3 Department of Pharmacology, Zhong-shan School of Medicine, Sun Yat-sen University, Guangzhou, China; College of Tropical Agriculture and Human Resources, University of Hawaii, United States of America

## Abstract

High-fat diet (HFD) is an environmental factor that contributes to the pathogenesis of obesity and type 2 diabetes. A number of genes influencing oxidative phosphorylation (OXPHOS) were found to be downregulated in skeletal muscle of humans and rats treated with HFD and have been implicated in mitochondrial dysfunction, insulin resistance, and consequent type 2 diabetes. In this study, we hypothesized that DNA methylation plays a crucial role in the regulation of OXPHOS genes in skeletal muscle of rats exposed to HFD. Using whole genome promoter methylation analysis of skeletal muscle followed by qPCR and bisulfite sequencing analysis, we identified hypermethylation of Cox5a in HFD rats. Furthermore, we found that Cox5a hypermethylation was associated with downregulation of Cox5a expression at the mRNA and protein level, and a reduction in mitochondrial complex IV activity and ATP content in HFD-induced insulin resistant rats compared to controls. Moreover, we found that while exposure to palmitate resulted in hypermethylation of the Cox5a promoter in rat myotubes, demethylation with 5-aza-2′-deoxycytidine was associated with preserved Cox5a expression, as well as restoration of complex IV activity and cellular ATP content. These novel observations indicate that Cox5a hypermethylation is associated with mitochondrial dysfunction in skeletal muscle of HFD-induced insulin resistant rats.

## Introduction

Type 2 diabetes mellitus (T2DM) is a heterogeneous and complex disease characterized by insulin resistance in adipose tissue, liver, and skeletal muscle, as well as impaired pancreatic insulin secretion. The etiology of insulin resistance and T2DM is multifactorial, involving both genetic and environmental factors [1], [2]. However, the mechanisms whereby genetic and environmental factors interact with each other in the development of T2DM still remain poorly understood. Epigenetic modifications are changes in gene function that occur without any alterations to the DNA sequence [3]. Accordingly, DNA methylation is an important example of epigenetic modification, often associated with downregulation of gene expression through methylation of cytosine sequences in the CpG islands of various promoter regions of DNA [4]. Notably, there is increasing evidence that DNA methylation is affected by environmental factors and hence, may be a potential molecular mechanism for the interaction between genetic and environmental factors in the development of obesity and T2DM [5]–[8]. Dietary intervention has been demonstrated to affect epigenetic modulation as reported, for example, in rats fed with a high-fat diet (HFD) during pregnancy [9], [10] and in agouti mice [11], [12]. Previous studies have also shown that acute exposure to free fatty acids (FFA) leads to DNA hypermethylation of the peroxisome proliferator-activated receptor γ (PPARγ) coactivator-1α (PGC-1α) promoter in myotubes of patients with T2DM [13]. Furthermore, hypermethylation of hepatic glucokinase (GCK) and L-type pyruvate kinase (LPK) promoters were found in HFD-induced obese rats and may be associated with insulin resistance [14]. The present evidence indicates that epigenetic modification by DNA methylation is a potential mechanism by which environmental factors interact with the epigenome, resulting in long-term changes in gene expression. However, it still remains unclear whether HFD exposure may induce epigenetic modification and how this may consequently lead to certain metabolic disorders such as obesity and T2DM.

Of note, oxidative phosphorylation (OXPHOS), a process that generates ATP from the proton gradient across the inner mitochondrial membrane, has been shown to be impaired in the skeletal muscle of people with T2DM and obesity [15]–[17]. Several groups have reported that the coordinated downregulation of OXPHOS genes in skeletal muscle of rats exposed to HFD may also contribute to mitochondrial dysfunction and the subsequent development of T2DM [18]–[22], though little is known about how exactly OXPHOS genes are regulated. Recently, however, some have argued for the role of epigenetic modification in the regulation of certain OXPHOS genes such as COX7A1 and NDUFB6, suggesting that acute reprogramming may play an important role in the development of T2DM [7], [23].

In the present study, we hypothesized that HFD exposure may lead to epigenetic modification of OXPHOS regulatory genes with subsequent downregulation of OXPHOS genes and mitochondrial dysfunction. We conducted a genome-wide promoter analysis of DNA methylation in skeletal muscle of HFD rats and demonstrated that hypermethylation of the Cox5a promoter was associated with concomitant mitochondrial dysfunction in skeletal muscle of HFD-induced insulin resistant rats.

## Materials and Methods

### Animal models

This study was carried out in strict accordance with the recommendation in the guide for the care and use of laboratory animals of the national institutes of health. All protocols were approved by the Animal Care Committee of Sun Yat-sen University (Permit Number: 2013050). Male Wistar rats (aged 4–5 weeks old) obtained from the Experimental Animal Center of Sun Yat-sen University (Guangzhou, China) were housed in a temperature-controlled room (22±2°C) and maintained on a 12-h light-dark cycle. These animals were randomly assigned to a standard chow diet (control) or a high-fat diet (HFD) of 60% kcal from fat (n = 7 per group; Research Diets Inc, New Brunswick, New Jersey, USA) for 16 weeks. Body weight was recorded weekly. After 16 weeks, intraperitoneal glucose tolerance test (IPGTT) was performed after 14 h of fasting. Rats were injected intraperitoneally with glucose at a dose of 2 g/kg body weight. Blood glucose was measured with glucose meter at 0, 15, 30, 60, 120 min (Roche Diagnostics, Mainz-Hechtsheim, Germany). Two days after the IPGTT test, insulin tolerance test was performed after 4 h of fasting. Rats were injected intraperitoneally with insulin at a dose of 0.75 U/kg body weight (Novolin-R, Novo Nordisk A/S). Blood glucose was measured with glucose meter at 15 min interval for 60 min.

Two days after insulin tolerance test, all rats were sacrificed by intraperitoneal injection of pentobarbital sodium (60 mg/Kg body weight) after 14 h of fasting. Plasma was separated by centrifugation and tested for total cholesterol, triglyceride, HDL, VLDL, free fatty acids (FFA) using an Architect Clinical Chemistry Autoanalyzer system. Plasma insulin was assayed using an insulin ELISA kit (Millipore,Billerica, MA,USA). Homeostasis model assessment (HOMA-IR) was calculated using the following equation: HOMA-IR  =  fasting glucose (mmol/L)×fasting insulin (mU/L)/22.5 [24]. The gastrocnemius muscle tissues were harvested and stored at −80°C for further analysis.

### Cell culture

Rat L6 skeletal muscle cells (Institute of Materia Medica, Shanghai, China) were grown in high glucose DMEM containing 4500 mg/L D-glucose, 10% fetal bovine serum (FBS), 100 U/ml penicillin, 100 µg/ml streptomycin (Invitrogen, Grand Island, NY, USA) until 40% confluent and then altered with differentiating media (2% FBS-DMEM) for 7–8 days [25]. Subsequently, myotubes were exposed to 0.2% BSA (control) or BSA-conjugated saturated fatty acid (0.4 mmol/L palmitate) (PA; Sigma, Saint Louis, MO, USA) in the presence or absence of 5 µM 5-aza-2′-deoxycytidine (5-Aaz-CdR; Sigma) for 72 h.

### MeDIP assay and microarray hybridization

Two gastrocnemius muscle tissues were randomly chosen from control group and HFD group. Genomic DNA (gDNA) was extracted using a DNeasy Blood & Tissue Kit (Qiagen, Fremont, CA, USA) and sonicated to random fragments of 200–1000 bp. Immunoprecipitation of methylated DNA (MeDIP) was performed using Biomag magnetic beads coupled mouse monoclonal antibody against 5-methylcytidine (Diagenode, Sparta, NJ, USA). The immunoprecipitated DNA was recovered with Proteinase K digestion followed by column-based purification (Qiagen), amplified using GenomePlex Complete Whole Genome Amplification kit (Sigma). The total input and immunoprecipitated DNA were labeled with Cy3- and Cy5-labeled random 9-mers, respectively, according to the manufacturer's guideline of the NimbleGen MeDIP-chip protocol (Nimblegen Systems, Inc., Madison, WI, USA) and hybridized to the NimbleGen Rat DNA Methylation 385K Promoter Plus CpG Island Array (Roche, Germany), which contains 15809 CpG Islands and gene promoter regions (from about −1300 bp to +500 bp of the TSSs) and totally covered by ∼385, 000 probes. Scanning was performed with the Axon GenePix 4000B microarray scanner.

### Analysis of microarray

Log2 ratio data were generated by performing median-centering and quantile normalization by Bioconductor packages Ringo and limma. From the normalized log2-ratio data, a sliding-window peak-finding algorithm provided by NimbleScan v2.5 was applied to find the enriched peaks with specified parameters (sliding window width: 750 bp; mini probes per peak: 2; p-value minimum cutoff: 2; maximum spacing between nearby probes within peak: 500 bp). A one-sided Kolmogorov-Smirnov (KS) test was applied to determine whether the probes were drawn from a significantly more positive distribution of intensity log2-ratios than those in the rest of the array. Each probe received a -log10 p-value score from the windowed KS test around that probe. When comparing differentially enriched regions between groups, we averaged the log2-ratio values for each group (HFD and control) and calculated the M′ value for each probe, where M′ = Average (log2 MeDIP_H_/Input_H_) - Average (log2 MeDIP_C_/Input_C_). The differential enrichment peaks (DEP) reported by the NimbleScan algorithm were included according to the following criteria: i) at least one of the two groups must have had a log2 MeDIP/Input> = 0.3 and M′>0; and, ii) at least half of probes in a peak must have had a coefficient of variability (CV) < = 0.8 overall in both groups.

### Bisulphite sequencing

Genomic DNA bisulphite modification was performed using Epitect Bisulfite Kit (Qiagen, Valencia, CA, USA) according to the manufacturer's instructions. The following primers were used for amplification of Cox5a promoter: sense 5′ TTA GTT TGT AGA GGG TTG GGA TTA TAG TA 3′; antisense5′ ACC ACA ACA CAC TAA CTA AAA CTA AAA A 3′. For Cox4i1: sense 5′ TGT AGT ATT ATT TTG TAG TAG GTT TGG GTG GTT AG 3′; antisense 5′ CTA TCA AAA ACC ATC ACC TAC CAC TAC T 3′. For amplification of the region from −253 to 0 of Cox5a promoter, the following primers were used: sense 5′ GTA GGA ATG TTT ATT ATA GTT AGT TAG GTT A 3′; antisense 5′ CAA CAC ACT AAC TAA AAC TAA AAA AAA ACC 3′. For Cox4i1 (−196, +101): sense 5′ GTT AAG ATG AGT TTT TAT TAT TAG TTA TAG TTT TT 3′; antisense 5′ TCA AAA ACC ATC ACC TAC CAC TAC TAC CCA AA 3′. PCR products were purified using MinElute Gel Extraction Kit (Qiagen) and cloned into PMD19-T vector (Takara, Dalian, China). For each condition, 10–15 clones were sequenced and the number of methylated sites was determined using Chromas software. The proportion of methylation for each individual was calculated by dividing the total number of methylated sites in all clones by the total number of CG sites.

### RT-PCR analysis

Total RNA was extracted from gastrocnemius muscles or cultured cells using TRIzol reagent (Invitrogen, Carlsbad, CA, USA). According to the manufacturer's protocol, cDNA was synthesized by reverse transcription using ReverTra Ace (Toyobo, Osaka, Japan). PCR was performed in a final volume of 10 µl consisting of diluted cDNA sample, primers, and SYBR Green Real-time PCR Master Mix (Toyobo) using a sequence detection system (ABI PRISM7900). The relative gene expression was calculated by 2^−△△CT^ method. PCR primer sequences are shown in Table S3.

### Western Blotting

Protein samples were extracted in RIPA lysis buffer containing protease inhibitors (Thermo Fisher Scientific, Waltham, MA, USA), then separated on 12% SDS-PAGE gels, electrophoretically transferred onto PVDF membranes (Millipore) for Western blotting analysis using anti-Cox5a antibody 1∶800 (11448-1-AP, Proteintech, Chicago, IL, USA); anti-GAPDH antibody, 1∶1000, (#2118, Cell Signaling Technology, Beverly, MA, USA). Membranes were washed twice in TBST and incubated in blocking buffer (5% non-fat milk in TBST) for 60 min at room temperature. Then membranes were washed three times and incubated overnight at 4°C with primary antibodies. Then the membranes were incubated with secondary antibodies (1∶1000, #7074, Cell Signaling Technology) for 1 h at room temperature and visualized by ECL detection (Pierce Biotechnology, Rockford, IL). Quantitation was performed using a Fujifilm Las-3000 Luminescent Image Analyzer.

### Determination of mitochondrial complex IV/cytochrome c oxidase (COX) activity

Complex IV activity was determined colorimetrically by following the oxidation of reduced cytochrome c as an absorbance decrease at 550 nm. The COX activity of tissue and cell extracts (50 µg/200 µl) was performed using the Rapid Microplate Assay kit for Rat complex IV activity (Mitosciences, Eugene, OR, USA) following the manufacturer's instructions.

### Determination of cellular ATP levels

Cellular ATP levels were measured using firefly luciferase-based ATP assay kit (Beyotime, China) according to the manufacturer's instructions. The concentration of the extracted proteins was determined using the Bradford Protein assay. ATP levels were determined by mixing 50 µl of the supernatant with 50 µl of luciferase reagent. The emitted light, which was linearly related to the ATP concentration, was measured using a multimode plate reader (Infinite F500, Tecan, Switzerland).

### Statistical analysis

Data are presented as mean±SD and all statistical analyses were performed using SPSS software (Version 13). Statistical analyses were performed using Student's t test, one-way ANOVA, and the Kruskal-Wallis test. The Pearson correlation was used to compare Cox5a methylation levels and Cox5a expression levels. p<0.05 was considered statistically significant.

## Results

### HFD causes obesity and insulin resistance in Wistar rats

**Figure 1:**
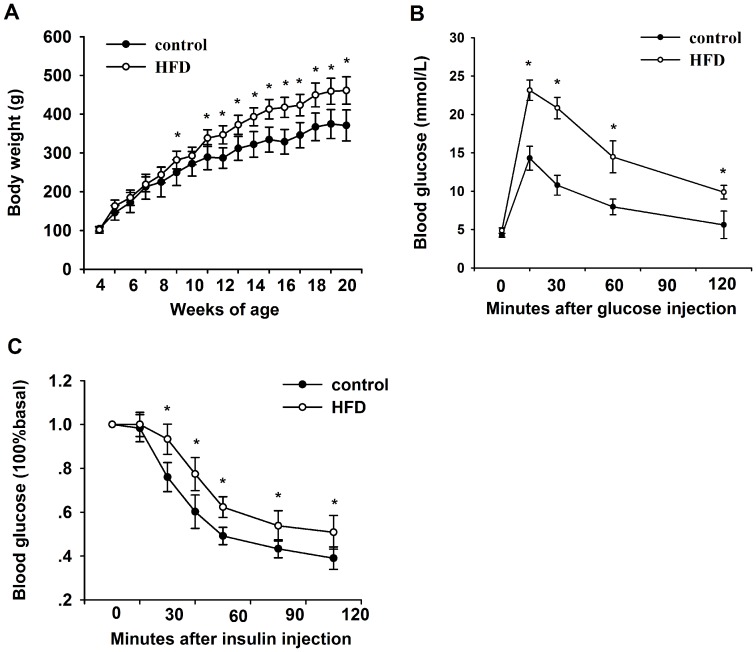
Body weight, glucose tolerance test and insulin tolerance test in HFD rats and control rats. (A) Body weight curves for Wistar rats fed on a standard rodent chow diet (control) or a high-fat diet (HFD) starting at 4 weeks of age. (B) Glucose tolerance test. (C) Insulin tolerance test. The baseline glucose levels are 5.5±0.3 mmol/L in control and 6.0±0.3 mmol/L in HFD group. Mean±SD. (n = 7/group. ANOVA, *p<0.05 vs control).

Wistar rats fed HFD had a significantly greater increase in mean body weight from week 7 to 16 (Figure 1A). We demonstrated that a significant difference of glucose tolerance still existed after 14 h of fasting in HFD rats compared with control rats (Figure 1B), although a previous study showed that longer fasting could enhance insulin sensitivity in mice [26]. As shown in Figure 1C, the insulin tolerance test results indicated that insulin resistance developed in HFD rats compared with control rats. By the end of 16 weeks, total cholesterol, TG, LDL and FFA levels were also higher among HFD rats (Table S1).

### Genome-wide analysis reveals differences in Cox5a promoter methylation

From the skeletal muscle obtained from the control and HFD rats, we identified 500 hypermethylated genes and 284 hypomethylated genes using MeDIP and microarray analysis. Functional analyses performed using the Kyoto Encyclopedia of Genes and Genomes (KEGG) revealed a differential distribution of genes across a broad range of metabolic pathways (Table S2).

**Figure 2:**
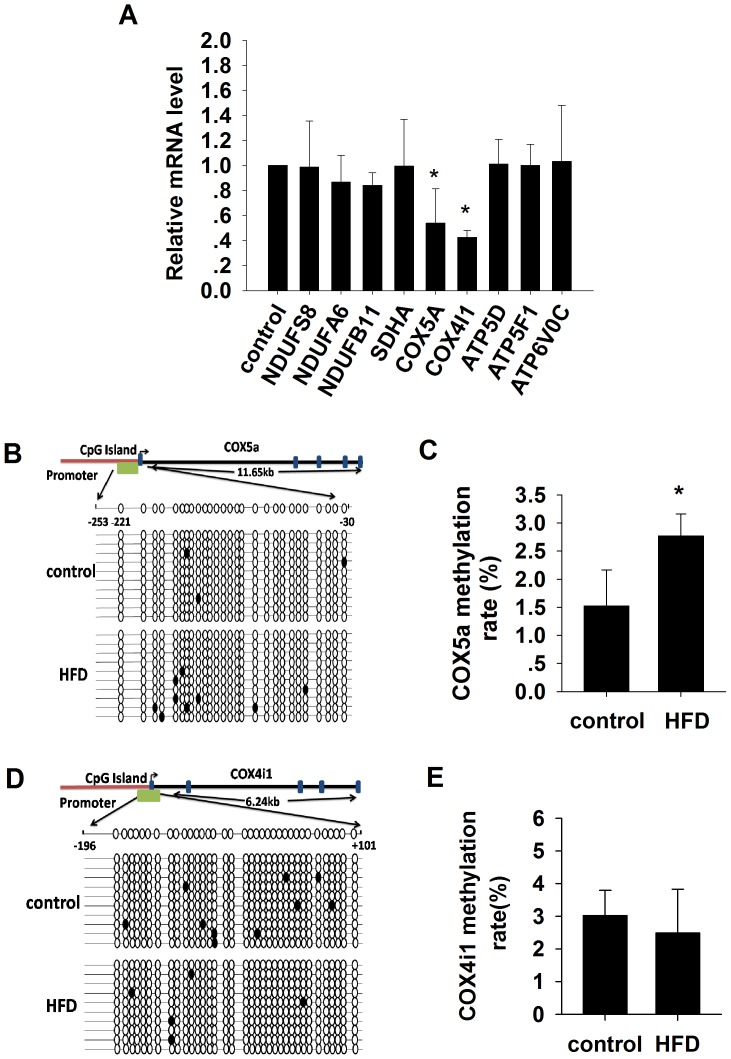
The mRNA levels of the 9 OXPHOS genes and methylation analysis of the Cox5a and Cox4i1 promoter by bisulfite sequencing. (A) Real-time PCR quantification of the 9 OXPHOS genes in skeletal muscle samples from control group and high-fat diet (HFD)-induced insulin resistant rat. Mean±SD. N = 4–7/group. *p<0.05 vs control. (B–C) Graphic representation and the results of the bisulfite-sequenced portion on the Cox5a gene. Mean±SD. (n = 7/group. ANOVA, *p<0.05 vs control). (D–E) Graphic representation and the results of the bisulfite-sequenced portion on the Cox4i1 gene. Mean±SD. (n = 7/group. ANOVA, *p<0.05 vs control).

Nine positive OXPHOS genes, all thought to be associated with mitochondrial dysfunction, were analyzed using real-time PCR. Significant reductions in the mRNA levels were found in the Cox5a and Cox4i1 genes but not the other genes in the HFD rats as compared to chow control (Figure 2A).

We performed bisulfite sequencing PCR amplification and found that the average methylation level for the Cox5a gene promoter was significantly higher in HFD rats compared to the control group (Figure 2B–C, Figure S1). There was, however, no significant difference observed for the Cox4i1 gene promoter (Figure 2D–E, Figure S2), suggesting that high-fat intake may selectively induce hypermethylation of Cox5a promoter in rat skeletal muscle.

### Downregulation of Cox5a mRNA expression and protein level correlates with promoter hypermethylation in skeletal muscle of HFD rats

**Figure 3:**
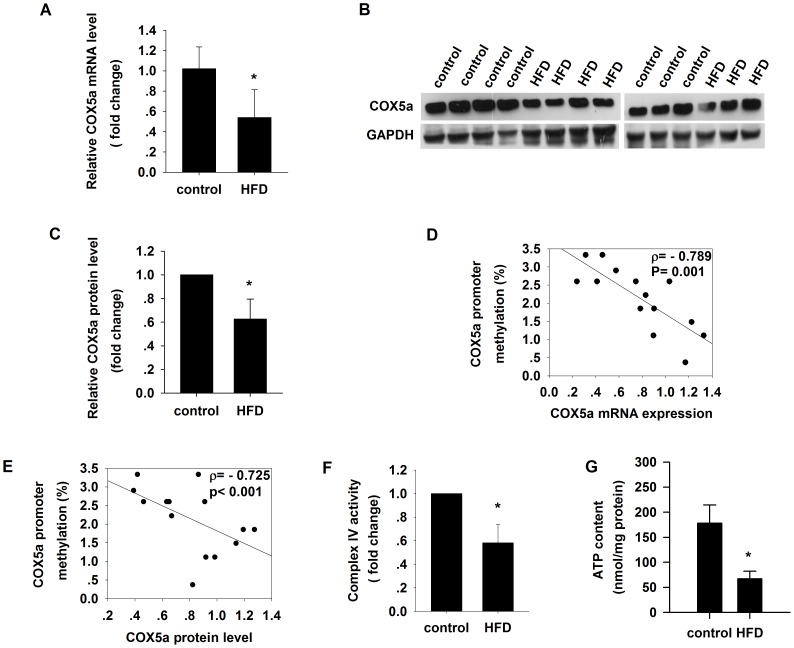
Cox5a promoter is hypermethylated in the skeletal muscle from high-fat diet (HFD)-induced insulin resistant rats. (A) Real-time PCR quantification of Cox5a mRNA expression in skeletal muscle from control and HFD groups. (B–C) Western blot of Cox5a protein level (n = 7/group). (D) Cox5a methylation levels are negatively correlated with Cox5a mRNA expression levels. (E) Cox5a methylation levels are negatively correlated with Cox5a protein levels. (F) Complex IV activity. (G) Cellular ATP content. Mean±SD. (n = 7/group. Student's t test, *p<0.05 vs control.)

We then determined whether downregulation of Cox5a gene expression (Figure 3A) was associated with changes in Cox5a protein level. We found lower levels of protein expression associated with Cox5a among HFD rats compared to control (Figure 3B–C). Accordingly, Cox5a promoter methylation was inversely correlated with both Cox5a mRNA expression (ρ = −0.789, p = 0.001; Figure 3D) and protein levels (ρ = −0.725, p<0.001; Figure 3E).

### Reduced mitochondrial complex IV activity and ATP content in skeletal muscle of HFD rats

As decreased expression of OXPHOS genes may result in mitochondrial dysfunction because of disruption in oxidative phosphorylation and ATP deprivation [27]–[30], we directly measured mitochondrial complex IV activity and found that HFD rats had significantly lower mitochondrial complex IV activity (Figure 3F) and decreased levels of cellular ATP (Figure 3G) as compared to control.

### Palmitate (PA) induces insulin resistance of L6 cells and hypermethylation of Cox5a promoter

**Figure 4:**
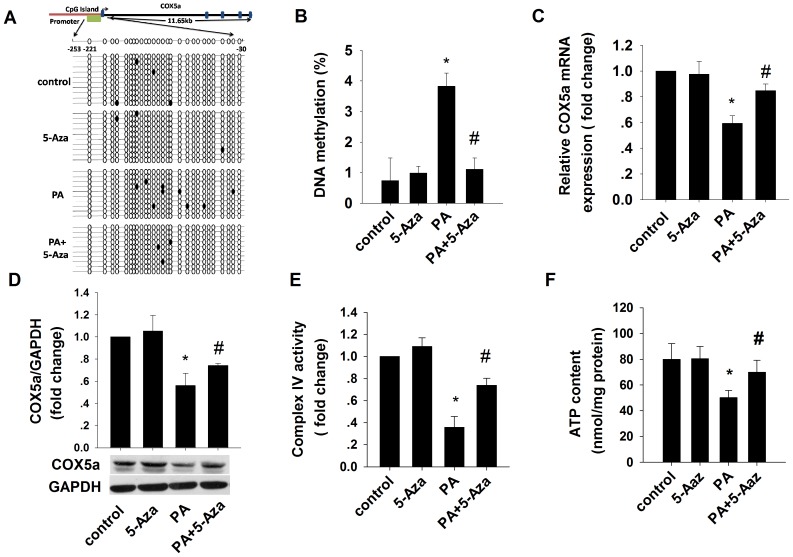
Effects of 5-Aza-CdR on DNA methylation levels of Cox5a promoters in palmitate (PA)-induced insulin-resistant L6 cells. Cells were treated with PA (0.4 mM), 5-Aza-CdR (5 µM) for 72 h, or both. (A–B) Graphic representation and the results of the bisulfite-sequenced portion on Cox5a gene. Results are mean±SD for 10 independent clones (ANOVA, *p<0.05 vs control; # p<0.05 vs PA). (C) Real-time PCR quantification of Cox5a mRNA expression. (D) Western blot of Cox5a protein level. (E) Complex IV activity. (F) ATP content. Mean±SD. n = 3. ANOVA, *p<0.05 vs control; # p<0.05 vs PA.

Elevated levels of FFA are thought to contribute to peripheral insulin resistance in obesity [31]. We found that treatment with 0.4 mM PA reduced glucose uptake and consumption in L6 cells (Figure S3) and it also downregulated mitochondrial complex IV activity and cellular ATP concentration in these cells (Figure 4E–F), suggesting that PA causes mitochondrial dysfunction and insulin resistance, in skeletal muscle cells.

To explore whether PA treatment alters Cox5a promoter methylation, we examined the impact of the methylation inhibitor 5-Aza-CdR on L6 cells selectively treated with PA. As shown in Figure 4A–B, hypermethylation of the Cox5a promoter was inhibited by 5-Aza-CdR.

In order to establish whether Cox5a methylation controls gene expression in L6 cells, we examined Cox5a mRNA expression using real-time PCR and found that the level of Cox5a mRNA was significantly reduced by PA treatment; the addition of 5-Aza-CdR blocked the effect of PA (Figure 4C). Similarly, Cox5a protein levels, which were reduced by PA treatment, was likewise restored by the presence of 5-Aza-CdR (Figure 4D). Taken together, these data suggest that DNA methylation may silence Cox5a expression.

Cox5a is one of the most important subunits in the core cytochrome c oxidase, a key holoenzyme in the mitochondrial respiratory chain. To determine whether mitochondrial function is altered in L6 cells by FFA, we further examined mitochondrial complex IV activity and cellular ATP content. Following PA treatment, we found that both complex IV activity and cellular ATP content were decreased, but these levels were restored by 5-Aza-CdR (Figure 4E–F), suggesting that DNA methylation may be involved in a PA-induced mitochondrial dysfunction pathway.

## Discussion

Lipid overload may impair mitochondrial oxidative capacity in skeletal muscle, potentially contributing to the pathogenesis of insulin resistance and T2DM [32]–[34]. In this study, we provided evidence for the relationship between HFD and the epigenetic modifications in skeletal muscle in rats as revealed by genome-wide screening of DNA methylation. We found that HFD led to hypermethylation of the promoter of the Cox5a gene. We also demonstrated that hypermethylation of the Cox5a promoter was associated with reduced Cox5a mRNA and protein expression, as well as reduced mitochondrial complex IV activity and cellular ATP content, providing a potential explanation for the mitochondrial dysfunction observed in skeletal muscle of HFD-induced insulin resistant rats.

Recent reports have demonstrated that DNA methylation effects may be a major molecular mechanism mediating dynamic gene-environment interactions contributing to the development of T2DM [7], [13], [14], [23], [35]. Selected reduction of mitochondrial OXPHOS genes expression is believed to impair the oxidative capacity of skeletal muscle in the setting of fat-induced insulin resistance [20]–[22], [36]. It is noteworthy that hypermethylation of the promoter for genes such as PGC-1α, COX7A1, and TFAM may be involved in mitochondrial function and insulin resistance [5], [7], [13]. This increase in DNA methylation was associated with decrease in gene expression. Given the present evidence, we propose that increased DNA methylation in mitochondrial OXPHOS genes may contribute to reduced gene expression and consequently impaired mitochondrial function.

Using genome promoter methylation analysis of skeletal muscle from HFD group and control group, we found that Cox5a was one of the genes that were hypermethylated after HFD feeding. Notably, Cox5a, a nuclear gene encoding cytochrome c oxidase subunit 5a, is crucial to the overall function of cytochrome c oxidase (COX) molecules in eukaryotic cells [28], [30], [37], [38]. COX catalyses the electron transfers from cytochrome c to oxygen, thereby contributing to energy storage across the electrochemical gradient. Accordingly, deficiency of the Cox5a results in severe mitochondrial dysfunction [28], [29]. We show that Cox5a promoter hypermethylation reduces Cox5a expression with concomitant reduction in mitochondrial complex IV activity and ATP content. Our findings suggest that lipid overload produces differential hypermethylation of the Cox5a promoter that may result in mitochondrial dysfunction, a novel observation that is consistent with and extends those of previous reports [5], [7], [13]. It is known that HFD and palmitate can impair insulin action through a variety of mechanisms, and that mitochondrial complex IV activity and ATP levels might be altered through additional pathways beyond the decreased expression of Cox5a observed in our study. PGC-1α is a master regulator of mitochondrial biogenesis and function. The PGC-1α promoter was found hypermethylated which was associated with its reduced expression in skeletal muscle from IGT and T2DM patients [13], [39]. Thus, PGC-1α may be another factor that impairs the HFD-induced mitochondrial function. Additionally, factors such as Cox7A1 and TFAM may also lead to mitochondrial dysfunction in insulin resistance [5], [7]. Nevertheless, our finding of the hypermethylation of Cox5a provides another example of how epigenetic factors affect mitochondrial function.

Previous evidence showed excessive FFA exposure might alter gene expression through epigenetic modifications [13], [14]. To corroborate our findings in rats, we treated rat L6 skeletal muscle cells with PA to determine the role of fatty acids in epigenetic modification of Cox5a mRNA expression. Our results showed that PA treatment resulted in DNA methylation and led to transcriptional silencing of the Cox5a gene. Furthermore, downregulation of Cox5a resulted in decreased complex IV activity and cellular ATP content, which are plausibly related to the pathogenesis of subsequent insulin resistance. There is increasing evidence that epigenetic modifications are subject to dynamic variations, much more than previously appreciated [40]. Acute FFA and TNF-α exposure, for example, has been shown to induce methylation at the PGC-1α promoter in human myocytes [13]. Correspondingly, our data demonstrate that FFA acutely induced the methylation of Cox5a promoter, indicating that this might be an early event in the pathogenesis of insulin resistance.

It is suggested that epigenetic modification may contribute to the development of T2DM [41], [42], as DNA methylation alters the expression of different genes like COX7A1, NDUFB6, PGC-1α and PPAR-δ, which are essential to normal mitochondrial function in skeletal muscle tissue [7], [13], [23], [40]. Moreover, changes in DNA methylation may also play an important role in the metabolic programming of pancreatic β-islet cells, glucose metabolism, and glucose transport [6], [14]. Our present study provides further evidence suggesting that HFD-induced differential hypermethylation of a specific OXPHOS regulatory gene may contribute to mitochondrial dysfunction and consequent insulin resistance and T2DM. The systematic profiling of DNA methylation secondary to HFD-induced insulin resistance may continue to yield valuable insights into the epigenetic mechanism of insulin resistance and T2DM in the future.

A potential weakness of our study is the lack of understanding of whether the changes in Cox5a expression are sufficient or necessary for insulin resistance in skeletal muscle or myotubes. However, the main objective of our study is to investigate whether hypermethylation of Cox5a is associated with mitochondrial dysfunction in skeletal muscle of high-fat fed rats, which might be a potential mechanism for HFD-induced insulin resistance. It will be interesting to further explore the link between mitochondrial dysfunction and insulin resistance in the future.

## Conclusions

In summary, HFD-induced hypermethylation of the Cox5a promoter in the skeletal muscle of rats was associated with downregulation of its mRNA and protein expression. FFA exposure with PA treatment in L6 cells was demonstrably associated with reduced mitochondrial complex IV activity and decreased levels of cellular ATP. These findings underscore a key role of HFD in epigenetic modification, resulting in altered gene expression and mitochondrial dysfunction, and highlight a potential pathway by which high-fat intake may contribute to the development of insulin resistance.

## Supporting Information

Figure S1 **Visualization of the Cox5a methylation results by bisulfite sequencing.**
(TIF)

Figure S2 **Visualization of the Cox4i1 methylation results by bisulfite sequencing.**
(TIF)

Figure S3 **Glucose metabolism in palmitate (PA)-induced L6 cells.** After treating with 0.4 mM PA for 72 h, the L6 cells were incubated in the presence or absence of 100 nm insulin for 30 min. (A) Glucose consumption, (B) glucose (2-NBDG) uptake by flow cytometry. Values are means ±SD for n = 3 experiments. Student's t test, *p<0.05 vs control.
(TIF)

Table S1 **Blood biomarkers from rats fed on a control diet or a high-fat diet (HFD) for 16 weeks.**
(DOCX)

Table S2 **Gene clustering of hypermethylated genes in HFD group from MeDIP array results by KEGG.**
(DOCX)

Table S3 **PCR primer sequences.**
(DOCX)
