# Supplementary figures and images for: Hypermethylation of Cox5a Promoter Is Associated with Mitochondrial Dysfunction in Skeletal Muscle of High Fat Diet-Induced Insulin Resistant Rats
Source: PLoS One. 2014 Dec 1;9(12):e113784. doi: 10.1371/journal.pone.0113784 (PMC4249960; doi:10.1371/journal.pone.0113784)

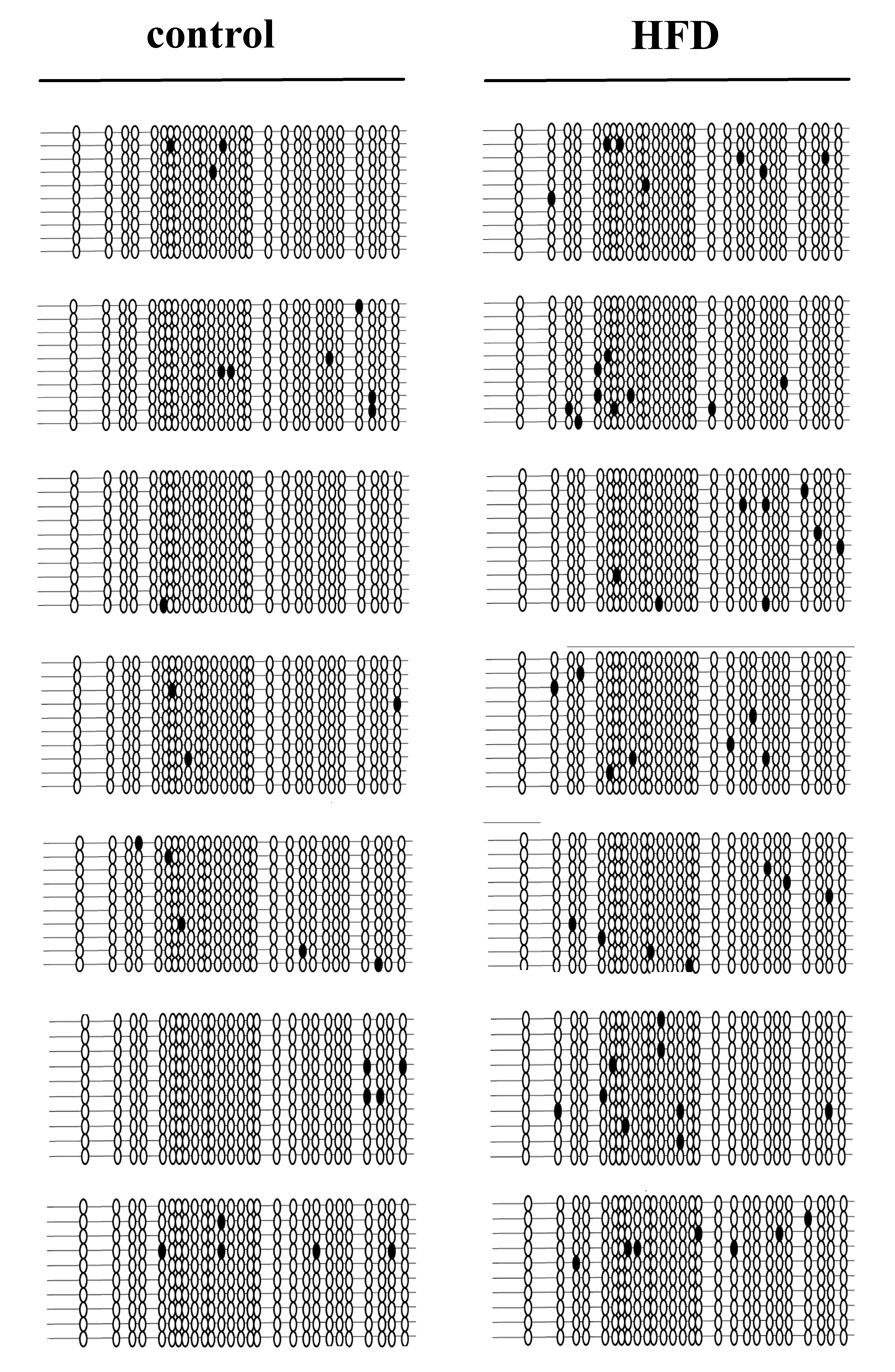

Supplement: Figure S1 — Visualization of the Cox5a methylation results by bisulfite sequencing. (TIF) [file pone.0113784.s001.tif]

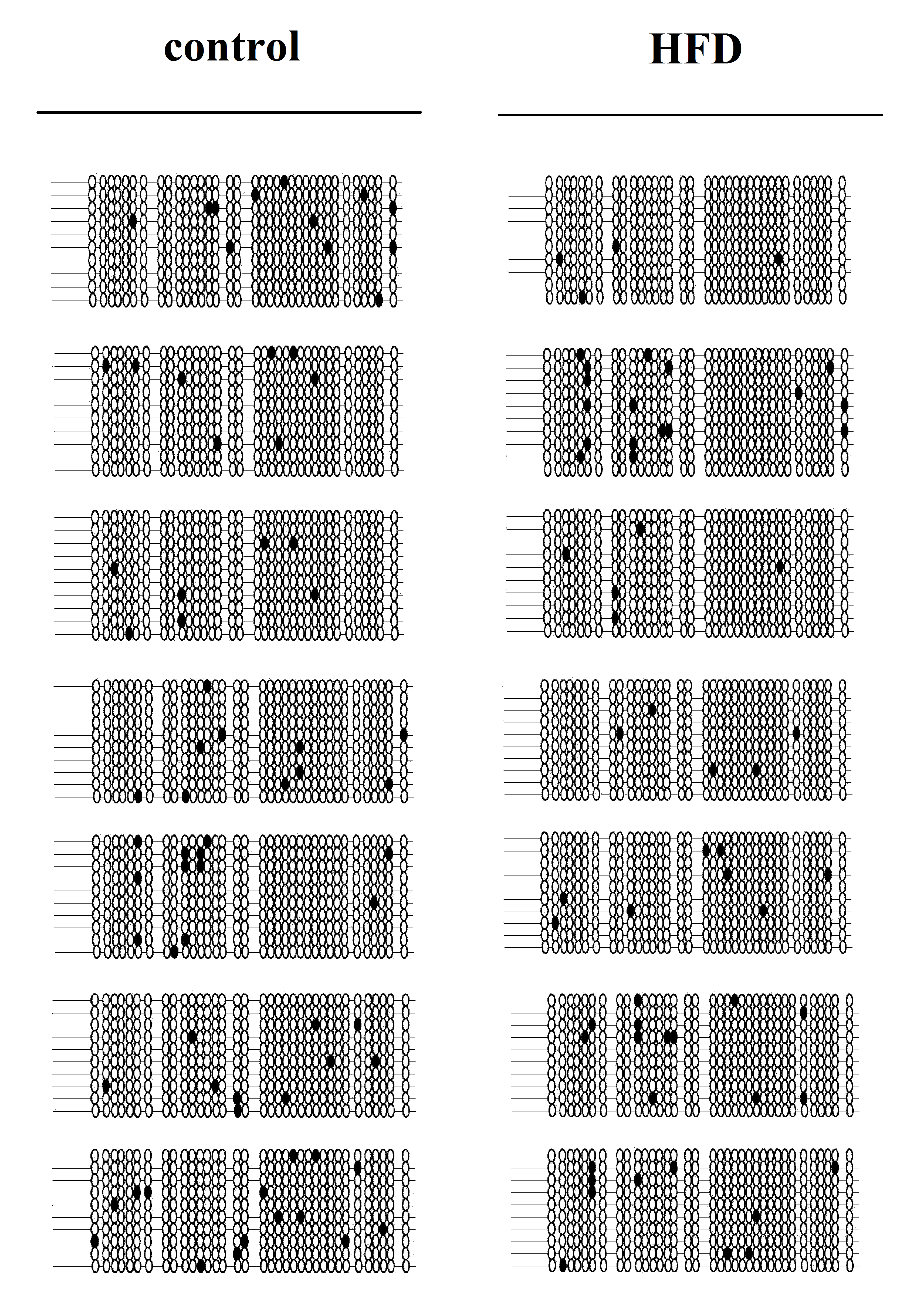

Supplement: Figure S2 — Visualization of the Cox4i1 methylation results by bisulfite sequencing. (TIF) [file pone.0113784.s002.tif]

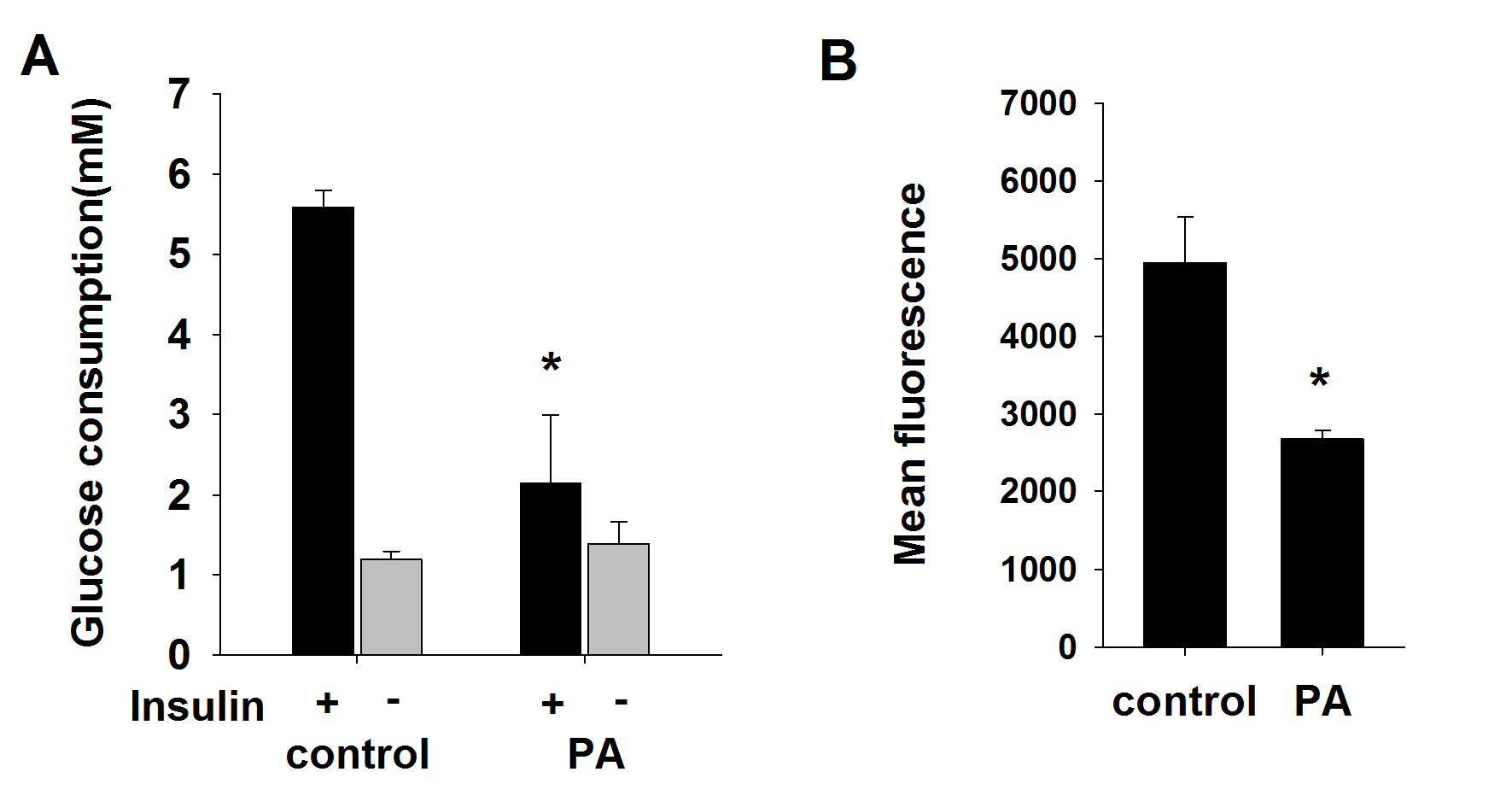

Supplement: Figure S3 — Glucose metabolism in palmitate (PA)-induced L6 cells. After treating with 0.4 mM PA for 72 h, the L6 cells were incubated in the presence or absence of 100 nm insulin for 30 min. (A) Glucose consumption, (B) glucose (2-NBDG) uptake by flow cytometry. Values are means ±SD for n = 3 experiments. Student's t test, *p<0.05 vs control. (TIF) [file pone.0113784.s003.tif]
