# Supplementary material for: Hypermethylation of Cox5a Promoter Is Associated with Mitochondrial Dysfunction in Skeletal Muscle of High Fat Diet-Induced Insulin Resistant Rats
Source: PLoS One. 2014 Dec 1;9(12):e113784. doi: 10.1371/journal.pone.0113784 (PMC4249960; doi:10.1371/journal.pone.0113784)
Supplement: Table S1 — Blood biomarkers from rats fed on a control diet or a high-fat diet (HFD) for 16 weeks. (DOCX) [file pone.0113784.s004.docx]

**Table S1. Blood biomarkers from rats fed on a control diet or a high-fat diet (HFD) for 16 weeks.**

| **Parameters** | **Control** | **HFD** |
| --- | --- | --- |
| **FPG（mmol/L）** | 5.45±0.34 | 5.98±0.33 |
| **FINS (ng/mL)** | 0.51±0.03 | 0.90±0.14* |
| **CHOL（mmol/L）** | 1.08±0.08 | 1.56±0.21 * |
| **TG（mmol/L）** | 0.34±0.07 | 0.74±0.36 ** |
| **HDL-c（mmol/L）** | 0.54±0.08 | 0.48±0.05 |
| **LDL-c（mmol/L）** | 0.22±0.04 | 0.29±0.09 * |
| **FFA（mmol/L）** | 0.38±0.35 | 0.58±0.06** |
| **HOMA-IR** | 3.06±0.24 | 5.87±0.98** |

Results are expressed as mean±SD (n = 7/group). *p<0.05; **p<0.01 versus control. FBG: fasting blood glucose; FINS: fasting insulin; CHOL: total cholesterol; TG: triglyceride; HDL: high density lipoprotein; LDL: low density lipoprotein; FFA: free fatty acid; HOMA-IR: homeostasis model assessment-insulin resistance.
