# Supplementary material for: Hypermethylation of Cox5a Promoter Is Associated with Mitochondrial Dysfunction in Skeletal Muscle of High Fat Diet-Induced Insulin Resistant Rats
Source: PLoS One. 2014 Dec 1;9(12):e113784. doi: 10.1371/journal.pone.0113784 (PMC4249960; doi:10.1371/journal.pone.0113784)
Supplement: Table S2 — Gene clustering of hypermethylated genes in HFD group from MeDIP array results by KEGG. (DOCX) [file pone.0113784.s005.docx]

**Table S2. Gene clustering of hypermethylated genes in HFD group from MeDIP array results by KEGG p<0.05.**

| **Pathway ID** | **Term** | **Counts** | **P-value** | **Enrichment Score** |
| --- | --- | --- | --- | --- |
| rno04725 | Cholinergic synapse | 11 | 0.000231 | 3.634765 |
| rno04142 | Lysosome | 11 | 0.000561 | 3.250702 |
| rno04122 | Sulfur relay system | 3 | 0.00211 | 2.675172 |
| rno04150 | mTOR-signaling pathway | 6 | 0.0028 | 2.552461 |
| rno04114 | Oocyte meiosis | 9 | 0.0042 | 2.378865 |
| rno04010 | MAPK signaling pathway | 14 | 0.0156 | 1.805636 |
| rno04110 | Cell cycle | 8 | 0.0212 | 1.673541 |
| rno04724 | Glutamatergic synapse | 8 | 0.0261 | 1.583123 |
| rno03430 | Mismatch repair | 9 | 0.0269 | 1.57032 |
| rno00190 | Oxidative phosphorylation | 9 | 0.0293 | 1.532615 |
| rno04962 | Vasopressin-regulated water reabsorption | 4 | 0.0317 | 1.499252 |
| rno04510 | Focal adhesion | 10 | 0.0347 | 1.460284 |
| rno04141 | Protein processing in endoplasmic reticulum | 9 | 0.0372 | 1.42892 |
| rno05212 | Pancreatic cancer | 5 | 0.0403 | 1.395092 |
| rno04727 | GABAergic synapse | 6 | 0.04195 | 1.377251 |
| rno04920 | Adipocytokine signaling | 5 | 0.0424 | 1.372282 |
| rno03420 | Nucleotide excision repair | 4 | 0.0474 | 1.324124 |
| rno04742 | Taste transduction | 4 | 0.0474 | 1.324124 |
| rno05032 | Morphine addiction | 6 | 0.0478 | 1.32099 |
| rno00020 | Citrate cycle (TCA cycle) | 3 | 0.0479 | 1.319619 |

**Pathway ID:** Pathway identifiers used in [KEGG](http://www.genome.jp/kegg/).

**Count:** Count of the chosen background population genes’ entities associated with the listed Pathway ID.

**p-value:** The enrichment p-value of the Pathway ID obtained using Fisher’s exact test.

**Enrichment Score:** The Enrichment Score value of the Pathway ID equals the average -log10 (p-value) from probes within the peak. The scores reflect the probability of positive enrichment.
