# Supplementary material for: Hypermethylation of Cox5a Promoter Is Associated with Mitochondrial Dysfunction in Skeletal Muscle of High Fat Diet-Induced Insulin Resistant Rats
Source: PLoS One. 2014 Dec 1;9(12):e113784. doi: 10.1371/journal.pone.0113784 (PMC4249960; doi:10.1371/journal.pone.0113784)
Supplement: Table S3 — PCR primer sequences. (DOCX) [file pone.0113784.s006.docx]

**Table S3. PCR primer sequences.**

| Name | Primer sequence(5'--3') | Accession No. |
| --- | --- | --- |
| NDUFS8 | F-CCACTGACCTCGTGAAGTCTTGT  R- CCCTTGACCTCTGACCCTATG | [NM_001106322.2](http://www.ncbi.nlm.nih.gov/nuccore/NM_001106322.2) |
| NDUFB11 | F-TTGTGAAATACCGAGAACTCAACG  R-CTGAGGAACCAGGAAATTAACAGAG | [NM_001106756.1](http://www.ncbi.nlm.nih.gov/nuccore/NM_001106756.1) |
| NDUFA6 | F-AAGAATGCCCATGTCACCGACCC  R-AATGTGTGTCCGCTGCTTCCAC | NM_001130505.1 |
| SDHA | F- ACACCGAATAAGAGCAAAGAACAC  R- AAATTCTAAGTCCTGGCAAGGT | [NM_130428.1](http://www.ncbi.nlm.nih.gov/nuccore/NM_130428.1) |
| Cox5a | F-CTGCCGCTGTCTGTTCCATTCG  R-TGTCACCCAGCGAGCATCAAACT | NM_145783.1 |
| Cox4i1 | F-TGGGCAGCAGTGGCAGAATGT  R-CCCGAAGGCACACCGAAGTAGA | NM_017202.1 |
| ATP5F1 | F-GAAGGCACAGCAGGCACTGGTT-3’  R-CCTCCAAGGCCAGGGCAATGTT-3’ | NM_134365.2 |
| ATP6V0C | F- CCACCACCAGTCACAGGATAGGA  R- AACAGACAATGGGCACTAGGACAC | [NM_130823.3](http://www.ncbi.nlm.nih.gov/nuccore/NM_130823.3) |
| ATP5D | F-GACACTGGACATGCTGGACCTCG  R-CTGTTACCCTGTGGGTGACAGAC | [NM_139106.1](http://www.ncbi.nlm.nih.gov/nuccore/NM_139106.1) |
| GAPDH | F-TGACTCTACCCACGGCAAGT  R-TACTCAGCACCAGCATCACC | [NM_017008.4](http://www.ncbi.nlm.nih.gov/nuccore/NM_017008.4) |
